# Inhibition of FOXM1 Leads to Suppression of Cell Proliferation, Migration, and Invasion Through AXL/eEF2 Kinase Signaling and Induces Apoptosis and Ferroptosis in GBM Cells

**DOI:** 10.3390/ijms26146792

**Published:** 2025-07-15

**Authors:** Ezgi Biltekin, Nermin Kahraman, Ogun Ali Gul, Yasemin M. Akay, Metin Akay, Bulent Ozpolat

**Affiliations:** 1 Department of Nanomedicine, Houston Methodist Research Institute, Houston, TX 77030, USA; ebilteki@central.uh.edu (E.B.);; 2 Department of Biomedical Engineering, University of Houston, Houston, TX 77004, USA; makay@central.uh.edu

**Keywords:** glioblastoma multiforme, eEF2K, FOXM1, AXL, gene regulation, apoptosis, ferroptosis, cancer stemness

## Abstract

Glioblastoma multiforme (GBM) is an aggressive and molecularly heterogeneous brain cancer with a poor prognosis. Despite advancements in standard-of-care therapies, including surgery, radiotherapy, and temozolomide (TMZ), the median survival remains approximately 15 months, with a 5-year survival rate of less than 10%. We and others have demonstrated that FOXM1 is a critical oncogenic driver of GBM cell proliferation. However, the role of FOXM1 and its interaction with other oncogenic signaling pathways in GBM remains incompletely understood. In this study, we identified FOXM1, AXL, and eEF2K as highly upregulated oncogenes in GBM patient tumors. We demonstrated, for the first time, that FOXM1 directly interacts with AXL and eEF2K, regulating their expression and promoting GBM cell proliferation, migration, and invasion. Knockdown of these genes disrupted cell proliferation, spheroid formation, migration, and invasion, and induced apoptosis and ferroptosis. Additionally, inhibiting the FOXM1–AXL/eEF2K signaling axis sensitized GBM cells to TMZ, further enhancing apoptotic and ferroptotic responses. These findings highlight the critical role of the FOXM1–AXL/eEF2K signaling pathway in GBM progression and suggest that targeting this axis may offer a novel multitargeted therapeutic strategy in GBM.

## 1. Introduction

Glioblastoma multiforme (GBM) is the most common and lethal primary malignant brain tumor in adults, comprising nearly half of all malignant brain tumors, and is recognized as an incurable brain cancer due to its molecularly heterogeneous and aggressive nature [1,2,3]. Standard treatment (surgery, radiotherapy, and TMZ) has modestly extended overall survival to approximately 15 months, but GBM remains essentially incurable. Additional FDA-approved modalities such as bevacizumab (an anti-angiogenic agent) and tumor treatment fields (TTFields) provide modest incremental benefits. Nonetheless, the 5-year survival remains < 10%, highlighting an urgent need for novel, multitargeted therapeutic strategies that address GBM’s profound molecular heterogeneity and drug resistance [1,2,4,5]. There is an urgent need for a better understanding of the complex biology and signaling pathways driving the proliferation and invasion of GBM cells to develop effective multitargeted therapies that can overcome the heterogeneous nature of GBM, which leads to aggressive and therapy-resistant phenotypes [6,7].

Forkhead-box protein M1 (FOXM1), a proto-oncogenic transcription factor, plays a role in tumor growth, resistance to chemotherapy, and stem cell maintenance, and its expression is associated with worse patient outcome. Although FOXM1 primarily functions as a transcription factor that drives the expression of genes implicated in cancer progression—such as those regulating the cell cycle, angiogenesis, and invasion—its activation requires cytoplasmic phosphorylation, predominantly mediated by ERK and cyclin-dependent kinases (CDKs). FOXM1 activity remains low during the G1/S transition and becomes markedly upregulated during the G2 phase, reflecting its critical role in cell cycle progression [8,9,10,11,12]. FOXM1 is also implicated in GBM pathogenesis, where it regulates stemness and therapy resistance to radiotherapy and chemotherapy and is associated with worse patient outcomes [13,14,15,16]. Despite its well-established multifunctional role in tumorigenesis, the precise molecular mechanisms driven by oncogenic FOXM1 signaling remain incompletely understood.

Eukaryotic elongation factor 2 kinase (eEF2K, eEF2-Kinase) is an oncogenic kinase overexpressed in various aggressive solid tumors, including triple-negative breast cancer, pancreatic, ovarian, and lung cancer. Its expression is associated with poor patient survival [17]. We and others have previously shown that eEF2K drives cell proliferation, migration, invasion, and tumor growth in triple-negative breast cancer, ovarian cancer, lung cancer, and pancreatic cancers [16,17,18,19,20,21].

AXL is a trans-membrane tyrosine kinase and a member of the TAM family [22]. It was first identified in leukemia patients, and subsequently it has been associated with tumorigenesis, drug resistance, immune response, and stemness in multiple cancers, including GBM [22,23,24,25,26]. Moreover, high expression of AXL is associated with poor patient survival, and the interaction of AXL with the tumor microenvironment and its role in immune regulation by involving macrophage polarization, T-cell function, and NK-cell activation shows its potential as a therapeutic target [26,27,28,29].

siRNAs (small interfering RNAs) that are approximately 21 nucleotide length single-strand RNAs, show their effect by base-by-base pairing and silencing the targeted gene [30]. They have been shown as effective tools to target GBM tumorigenesis by regulating oncogenes and promoting antitumor efficiency of chemotherapy in in vitro studies [16,31,32]. Moreover, siRNA-based therapies that are currently under FDA-approved clinical investigations have been employed for several different diseases since 2018 [33].

Overall, while we and others demonstrated FOXM1 functions as an oncogenic transcription factor and a critical driver of cell proliferation, its role as a cytoplasmic protein and the proteins that FOXM1 interact with, as well as downstream signaling in GBM is poorly understood.

In this study, we investigated the molecular mechanisms driven by FOXM1 and its role in regulating other oncogenic signaling pathways, such as AXL and eEF2K and their roles in mediating GBM proliferation and progression. We found that FOXM1, AXL, and eEF2K are significantly overexpressed in GBM patient tumor samples and FOXM1 regulates the expression of AXL and eEF2K by physically interacting with these proteins. Notably, inhibition of the FOXM1-AXL/eEF2K signaling axis led to significant suppression of cell proliferation, spheroid formation, migration, and invasion, and enhances temozolomide (TMZ)-mediated cell death by triggering both apoptosis and ferroptosis. These findings support the notion that targeting FOXM1-AXL/eEF2K could offer a multitargeted therapeutic strategy for GBM and may enhance the efficacy of TMZ.

## 2. Results

### 2.1. eEF2K, AXL, and FOXM1 Are Upregulated in GBM Patient Tumors

**Figure 1:**
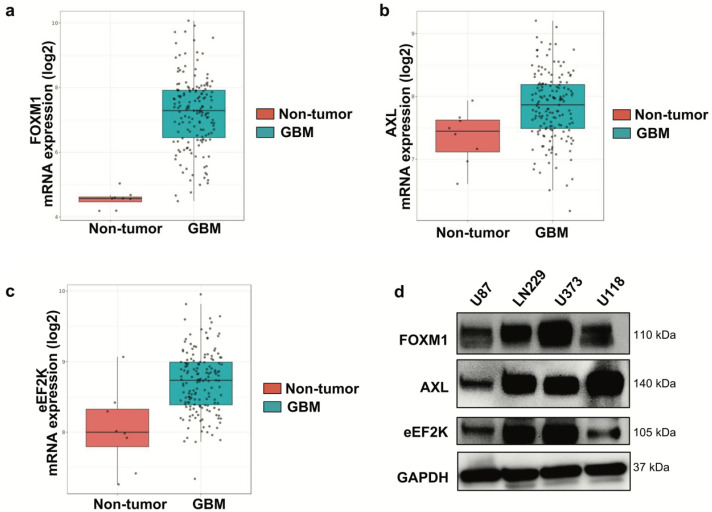
*FOXM1, AXL, and eEF2K are highly expressed in GBM tumor samples*. (**a**–**c**) The Gravendeel dataset was analyzed, showing that FOXM1 (**a**), AXL (**b**), and eEF2K (**c**) are highly expressed in GBM tumor samples compared to non-tumor samples. Data are presented as mRNA expression (log2). (**d**) Western blot analysis of GBM cell lines (LN229, U87, U373, U118) revealed expression of FOXM1, AXL, and eEF2K in all four cell lines at varying levels.

To evaluate the clinical relevance of FOXM1, AXL, and eEF2K in GBM, we analyzed the Gravendeel GBM patient dataset. All three genes were significantly upregulated in GBM tumors compared with non-tumor brain tissue (Figure 1a–c). Consistent with patient data, FOXM1, AXL, and eEF2K were expressed across four GBM cell lines, including LN229, U87, U373, and U118, though at varying levels (Figure 1d).

### 2.2. Downregulation of FOXM1, AXL, and eEF2K Inhibits Proliferation and Colony Formation

**Figure 2:**
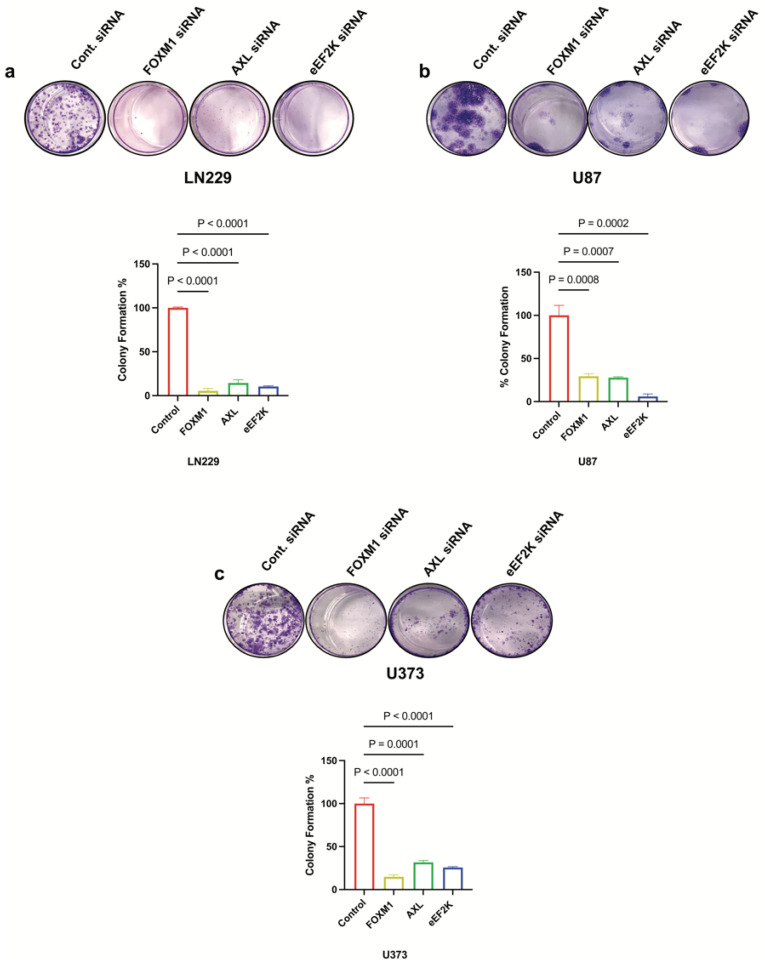
*FOXM1, AXL, and eEF2K downregulation through siRNA suppresses cell proliferation of GBM cells*. (**a**) Clonogenic assay results showing that downregulation of FOXM1, AXL, and eEF2K via siRNA significantly suppressed colony formation in LN229 cells treated with 25 nM siRNA (*p* < 0.0001). (**b**) Colony formation was significantly suppressed in U87 cells transfected with 25 nM siRNA targeting FOXM1, AXL, and eEF2K (*p* = 0.0008, *p* = 0.0007, *p* = 0.0003). (**c**) U373 colony formation was significantly suppressed by siRNA-mediated inhibition of FOXM1, AXL, and eEF2K (*p* < 0.0001, *p* = 0.0001, *p* = 0.0001).

To investigate the effects of FOXM1, AXL and eEF2K on cell proliferation and colony formation in GBM cells, we knocked down FOXM1, AXL, and eEF2K using specific siRNAs in different GBM cell lines (LN229, U87, and U373). We found that knockdown of FOXM1, AXL, and eEF2K through siRNA significantly suppressed cell proliferation and colony formation in all three cell lines (Figure 2a–c). These results suggest that all three oncogenic factors play integrated roles in GBM cell growth.

### 2.3. FOXM1 Regulates AXL and eEF2K Expression and Physically Interacts with Them

**Figure 3:**
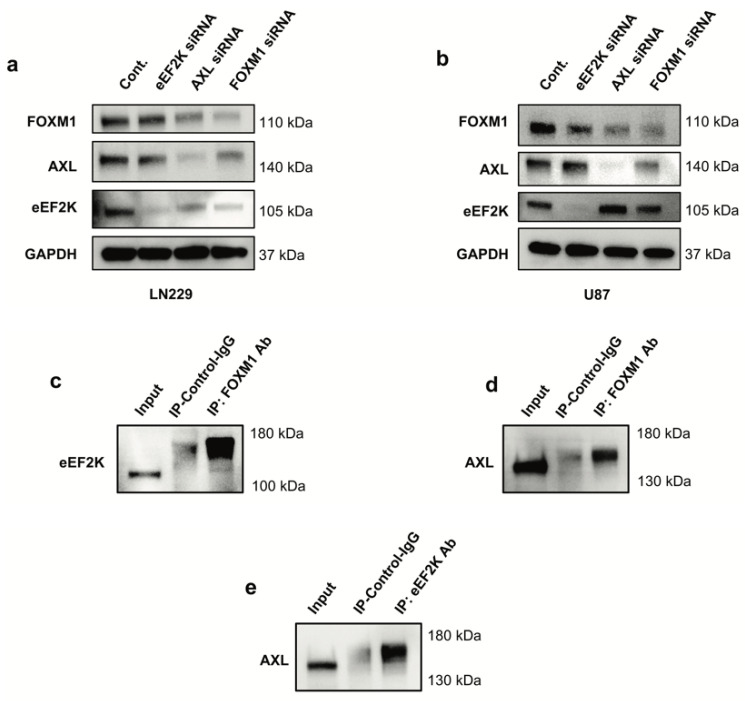
*Knockdown of FOXM1 by siRNA leads to reduced expression of AXL and eEF2K*. (**a**,**b**) LN229 and U87 cells were transfected with 100 nm for 72 h with control or specific siRNAs targeting FOXM1, AXL and eEF2K and protein expressions were analyzed by Western blotting. (**c**–**e**) Co-immunoprecipitation assays demonstrated the interaction between FOXM1, eEF2K, and AXL. FOXM1 forms heterodimers with eEF2K and AXL; eEF2K forms a heterodimer with AXL.

Western blot analyses revealed that siRNA-mediated downregulation of any one of these proteins decreased the expression of the others, suggesting cross-regulation (Figure 3a,b). The siRNAs used for targeting each oncogene effectively suppressed their corresponding gene expression. The inhibition efficiency of each siRNA analyzed (Figure S1) showed that all siRNAs reduced the expression of their target oncogenes by less than 30%. FOXM1 inhibition was the most effective in both cell lines for downregulating both AXL and eEF2K expression. In contrast, eEF2K inhibition primarily affected its own expression, suggesting that FOXM1 may function upstream in this regulatory network (Figure 3a,b). Co-immunoprecipitation assays confirmed physical interactions among FOXM1, AXL, and eEF2K (Figure 3c–e), supporting the existence of a higher-order FOXM1–AXL/eEF2K complex. FOXM1 did not only regulate the expression of AXL and eEF2K but also physically interacted with these proteins. We also demonstrated the interaction between AXL and the eEF2K protein. To our knowledge, this is the first demonstration of these molecules creating a heterodimer complex in GBM.

### 2.4. Inhibition of FOXM1, AXL, and eEF2K Suppresses Spheroid Formation of GBM Cells

**Figure 4:**
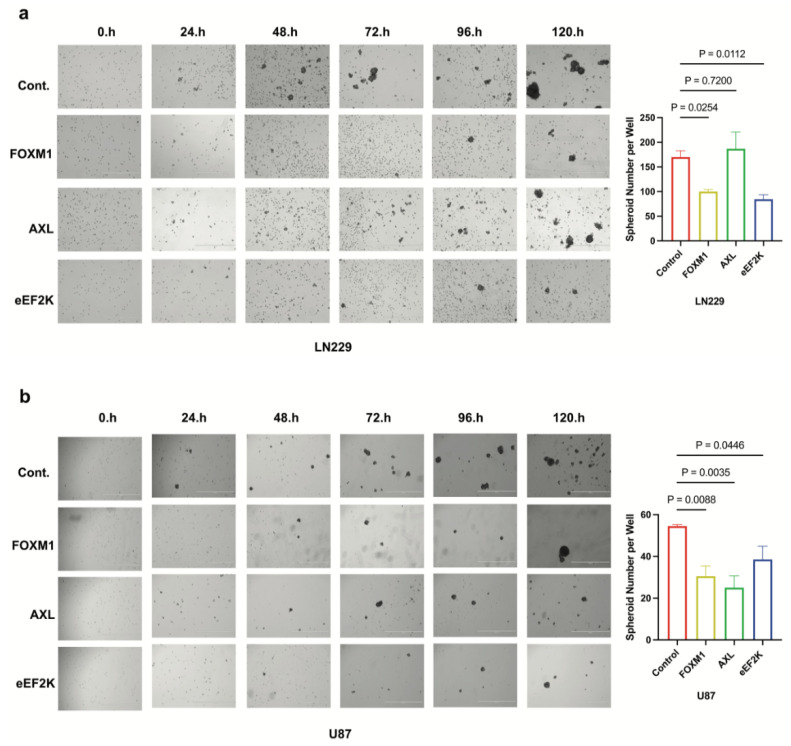
*FOXM1, AXL, and eEF2K inhibition through siRNA suppresses spheroid formation in GBM cells*. (**a**) LN229 cells transfected with siRNA targeting FOXM1, AXL, and eEF2K were seeded into ultra-low attachment plates as single cells, and spheroid formation ability was observed over five days. Decreased spheroid numbers were observed in FOXM1 (*p* = 0.0254) and eEF2K (*p* = 0.0112) downregulated cells at the end of day 5. (**b**) FOXM1, AXL, and eEF2K downregulation inhibited spheroid formation in U87 cells (*p* = 0.0088, *p* = 0.0035, *p* = 0.0446).

In non-adherent conditions, the ability of cells to form and grow as spheroids is a hallmark of stem-like properties. Thus, we assessed whether FOXM1 and its downstream mediators (AXL or eEF2K) are required for this phenotype. Knockdown of each gene decreased spheroid number and size, with eEF2K and FOXM1 siRNA showing the most consistent effect across both LN229 and U87 cell lines (Figure 4a,b) (FOXM1: *p* = 0.0254, *p* = 0.0088; eEF2K: *p* = 0.0112, *p* = 0.0446). Interestingly, AXL inhibition significantly reduced spheroid formation in U87 cells (*p* = 0.0035) but had no effect in LN229 cells (Figure 4a,b), highlighting potential cell line-specific features. eEF2K stands out as a central regulator of spheroid formation in both cell lines, acting both independently of and in connection with FOXM1.

### 2.5. Knockdown of FOXM1, AXL, and eEF2K Suppresses Cell Migration and Invasion of GBM Cells

**Figure 5:**
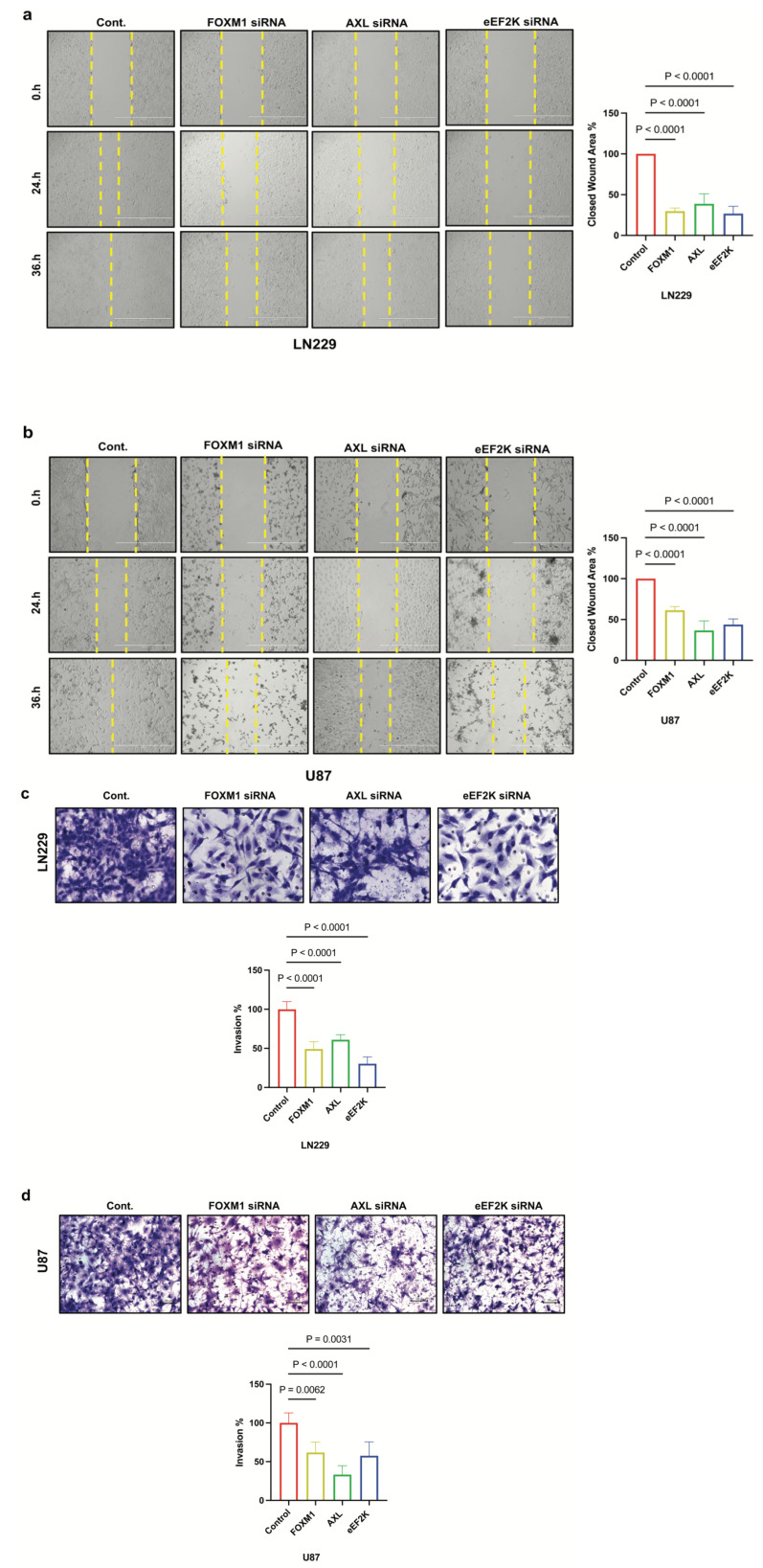
*FOXM1, AXL, and eEF2K inhibition through siRNA suppresses cell migration and invasion*. (**a**,**b**) LN229 and U87 cells transfected with 100 nM siRNA targeting FOXM1, AXL, and eEF2K for 72 h were subjected to wound-healing assays. Downregulation of FOXM1, AXL, and eEF2K significantly suppressed the migration ability of both LN229 and U87 cells (*p* < 0.0001). (**c**,**d**) LN229 and U87 cells transfected with 100 nM siRNA targeting FOXM1, AXL, and eEF2K for 72 h were seeded into Matrigel-coated chambers to assess invasion. Inhibition of FOXM1, AXL, and eEF2K suppressed the invasion ability of both LN229 and U87 GBM cells.

We investigated the role of FOXM1, AXL, and eEF2K in the metastatic potential and cell motility of GBM cell lines using a cell migration assay and a Matrigel-coated transwell invasion assay to assess cell invasion. We found that downregulation of FOXM1, AXL, and eEF2K by siRNA suppressed the migration and invasion abilities of LN229 and U87 GBM cells (Figure 5a–d). These data align with previous reports suggesting that all three molecules independently promote metastatic behavior in other cancers.

### 2.6. Downregulation of FOXM1, AXL, and eEF2K Induces Apoptosis and Ferroptosis

**Figure 6:**
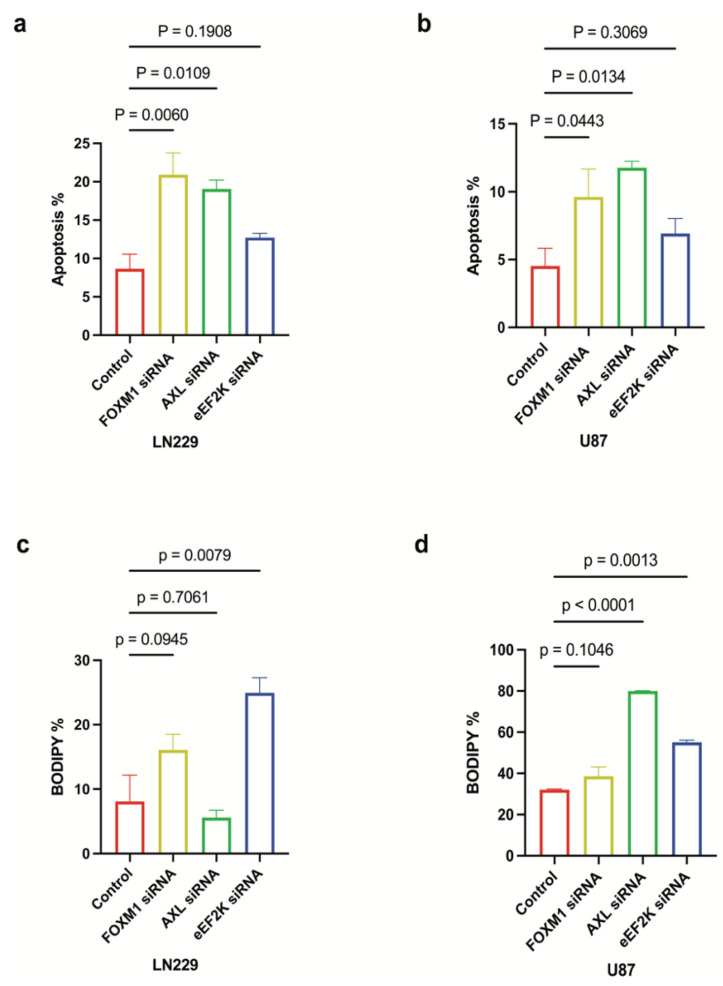
*FOXM1, AXL, and eEF2K inhibition through siRNA induces apoptosis and ferroptosis*. (**a**,**b**) LN229 and U87 cells transfected with 100 nM siRNA targeting FOXM1, AXL, and eEF2K for 96 h were analyzed for apoptosis using the Annexin V assay. Downregulation of AXL and FOXM1 induced apoptotic cell death in both cell lines. (**c**,**d**) LN229 and U87 cells transfected with 100 nM siRNA targeting FOXM1, AXL, and eEF2K for 96 h were analyzed for lipid peroxidation using a lipid peroxidation assay. eEF2K inhibition induced lipid peroxidation, which is a hallmark of ferroptosis.

To assess the effect of FOXM1, AXL, and eEF2K on GBM cell survival, we investigated cell death mechanisms after knocking down FOXM1, eEF2K, and AXL by specific siRNAs. Annexin V/propidium iodide assays indicated that AXL and FOXM1 knockdown induced significant apoptosis in LN229 and U87 cells’ induction (LN229: *p* = 0.0109, *p* = 0.0134) (U87: *p* = 0.0060, *p* = 0.0443), whereas eEF2K knockdown showed more modest apoptotic induction in LN229 (*p* = 0.1908) and U87 (*p* = 0.3069) cell lines (Figure 6a,b). From that point, we investigated the effect of FOXM1, AXL, and eEF2K inhibition on a distinct form of cell death, ferroptosis [34]. Lipid peroxidation is the hallmark of ferroptosis, and to show the effect of FOXM1, AXL, and eEF2K inhibition, we used BODIPY™ 581/591 C11 staining. Interestingly, eEF2K knockdown instead strongly induced lipid peroxidation, suggesting ferroptosis (Figure 6c,d). These findings underscore the diversity of the death pathways engaged when different components of this complex are downregulated.

### 2.7. Knockdown of FOXM1, AXL, and eEF2K Enhances TMZ-Induced Cell Death via Apoptosis and Ferroptosis

**Figure 7:**
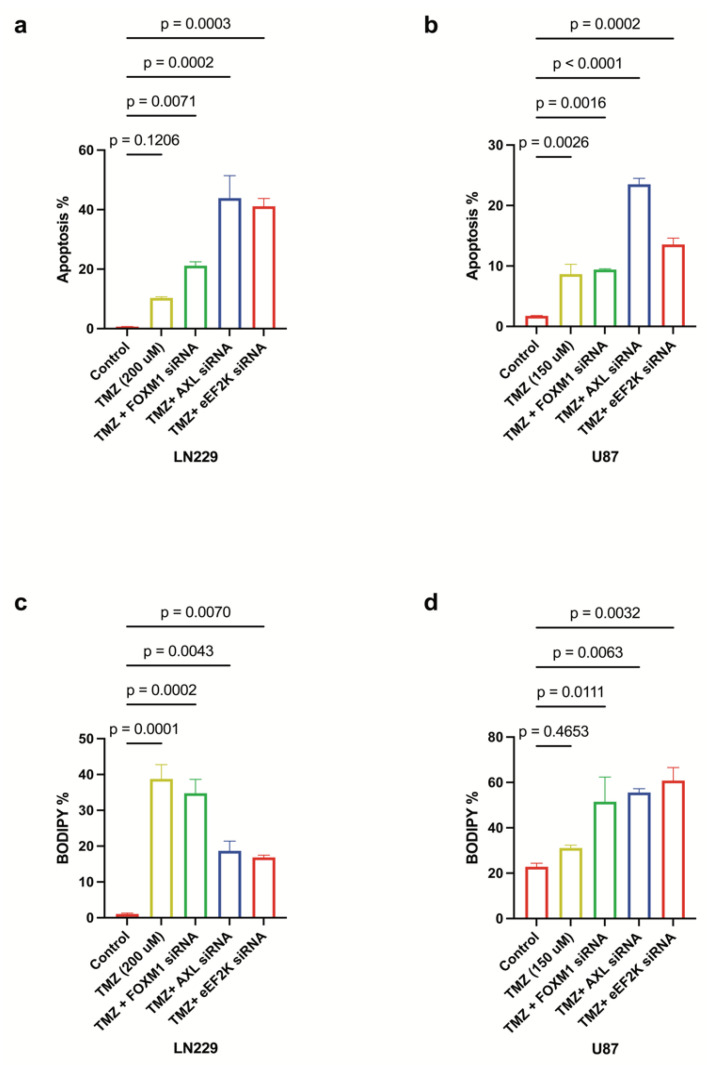
*FOXM1/AXL-eEF2K knockdown enhances the effects of TMZ chemotherapy by inducing both apoptosis and ferroptosis*. (**a**,**b**) LN229 and U87 cells transfected with 100 nM siRNA targeting, 24 h after siRNA transfection. FOXM1, AXL and eEF2K transfected cells were treated with TMZ (LN229: 200 uM, U87: 150 uM) for 72 h. Lipid peroxidation assays performed at 96 h showed that FOXM1, AXL, and eEF2K downregulation in combination with TMZ enhanced apoptotic cell death in both cell lines. (**c**,**d**) Lipid peroxidation assays performed at 96 h demonstrated that eEF2K and AXL downregulation in LN229 enhanced apoptosis in combination with TMZ, while eEF2K, AXL and FOXM1 downregulation in U87 predominantly induced lipid peroxidation, indicative of ferroptosis.

TMZ is a first-line chemotherapeutic for GBM, yet resistance is common: about 50% of GBM patients do not respond to TMZ treatment or develop resistance during treatment, contributing to poor patient survival [35]. We found that simultaneous knockdown of FOXM1, AXL, and eEF2K substantially augmented TMZ-induced cell death in LN229 and U87 cells (Figure 7a–d). Notably, in LN229 cells, eEF2K and AXL silencing reinforced apoptotic cell death in combination with TMZ (Figure 7a), while in U87 cells, enhanced ferroptosis was the predominant mechanism. These data highlight the benefit of combinatorial targeting of these oncogenes to overcome TMZ resistance and maximize GBM cell death.

## 3. Discussion

Glioblastoma multiforme is a highly malignant primary brain tumor, and patients with GBM typically have an overall survival time of approximately 15 months following diagnosis when treated with conventional therapeutic approaches, such as radiotherapy, surgery, and chemotherapy [1,6].

Despite ongoing advances in GBM therapy, patient prognosis remains poor, reflecting the disease’s exceptional molecular heterogeneity and invasive features. Therapeutic strategies that target single pathways often fail to achieve durable responses [36,37,38]. Our study provides novel insight into how FOXM1 mediates its oncogenic signaling in GBM cells where FOXM1 regulates AXL and eEF2K, both of which orchestrate GBM proliferation, invasiveness, and therapeutic resistance. More importantly, our data suggest that FOXM1, AXL, and eEF2K are clinically significant molecules and are overexpressed in GBM patient tumors.

**Figure 8:**
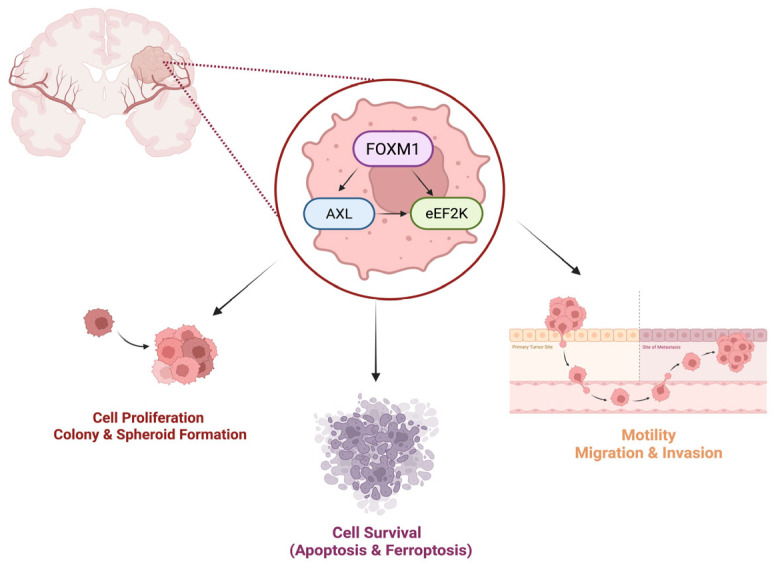
*eEF2K, AXL, and FOXM1 form heterodimers in GBM, and their downregulation suppresses cell proliferation, migration, invasion, and spheroid formation while inducing apoptotic cell death and ferroptosis*. Figure created in BioRender. Biltekin, E. (2025) https://BioRender.com/rflvw5x, accessed on 1 July 2025.

Our data suggest that FOXM1 regulates AXL and eEF2K, which play a significant role in promoting cell proliferation, survival, migration, invasion, and the spheroid formation ability of GBM cells. Interestingly, our studies revealed that these oncogenic molecules interact with each other and form a complex by binding to each other. Targeting FOXM1 reduced AXL and eEF2K expression, suggesting that FOXM1-targted therapies may offer multitargeting potential due to suppressing AXL/eEF2K signaling, leading to the inhibition of cell proliferation, cell migration–invasion, and spheroid formation ability and causing apoptosis and ferroptosis induction in GBM cells (Figure 8). Since FOXM1, AXL, and eEF2K have been linked to drug resistance, we also combined the first-line standard chemotherapeutic agent TMZ with FOXM1, AXL, and eEF2K-targeted strategies. Our studies indicate that a FOXM1-, AXL-, and eEF2K-targeted approach can sensitize GBM tumors to TMZ treatment. However, future studies should test this concept in in vivo orthotopic GBM tumor models.

eEF2K is an oncogenic kinase known for promoting cell proliferation, invasion, and migration in various cancers [17]. Previously, studies have shown that eEF2K is overexpressed in glioblastoma multiforme and plays a role in cell migration, invasion, and apoptosis in combination with temozolomide [33,34,39]. While our findings support the findings of Liu et al. [40] showing that eEF2K alone promotes cell migration and invasion and its inhibition promotes apoptosis, in contrast to their findings, we showed that its genetic inhibition suppresses cell proliferation in three different GBM cell lines. Also, we showed, for the first time, the regulatory role of eEF2K in spheroid formation, indicating its potential role in GBM stemness and drug resistance caused by stemness. When we analyzed GBM cells that were treated with eEF2K siRNA, we could not observe significant apoptosis induction, which led us to investigate the role of eEF2K on other cell death mechanisms. We showed for the first time that eEF2K inhibition induces lipid peroxidation, a key feature of ferroptosis in GBM cells.

On the other hand, AXL is another emerging potential therapeutic target in various cancers due to its effect in promoting cell proliferation, invasion and migration, and tumor growth [41]. In agreement with our study, researchers have previously found that AXL is overexpressed in glioblastoma cells and have shown that its inhibition induces apoptosis in vivo and suppresses cell migration and invasion [25,42,43,44]. We also showed that AXL inhibition leads to the suppression of spheroid formation specifically in the U87 cell line. U87 cells express significantly less AXL compared to LN229 cells. More importantly, studies have also reported differences in stemness marker expressions between LN229 and U87 spheroids. LN229 cell line spheroids expressed Nestin, Sox2, and Musashi-1, whereas U87 spheroid did not, which may contribute to the higher resistance observed in LN229 cells. Inhibition of AXL in LN229, the differential expression of stemness markers, and the presence of functional p53 and PTEN may trigger compensatory signaling pathways, allowing the cells to maintain survival and resistance [45,46]. This highlights the molecular background in the determination of the therapeutic approaches.

As an oncogenic transcription factor, FOXM1 drives the expression of many important signaling molecules that regulate the cell cycle, proliferation, invasion, and metastasis, making it an excellent molecular target in GBM, considering its significant heterogeneity. Therefore, targeting FOXM1 can prevent tumor progression and growth by suppressing key proteins involved in cell proliferation, invasion, angiogenesis, and tumorigenicity [16,47]. We also found that FOXM1 genetic inhibition leads to the inhibition of cell proliferation, migration, invasion, and spheroid formation while inducing apoptosis in GBM cells. Previously, our group showed the binding interaction between eEF2K and FOXM1 in triple negative breast cancer [9,48]. Furthermore, we identified for the first time the binding interaction between FOXM1, AXL, and eEF2K within the glioblastoma concept.

Our study also revealed, for the first time, an intricate and complex interaction and regulation between FOXM1, AXL, and eEF2K, which are clinically important oncogenic signaling pathways. We observed that the downregulation of AXL and FOXM1 suppresses the expression of eEF2K, AXL, and FOXM1 in GBM cell lines, suggesting potential direct interactions between these molecules. Our co-immunoprecipitation assay findings demonstrated, for the first time, that FOXM1, AXL, and eEF2K form heterodimer complexes in GBM cells. These findings also suggest that targeting the FOXM1-AXL/eEF2K interaction could stabilize their expression, and targeting each of these proteins may serve as an effective multitargeted therapeutic strategy. Considering its role in transcription, FOXM1 may act as the conductor in this interaction.

## 4. Conclusions

In conclusion, our study identifies FOXM1-AXL/eEF2K signaling as a mediator of GBM tumorigenesis. FOXM1 physically interacts with AXL/eEF2K and regulates these oncogenic factors that converge to form a previously unrecognized heterodimer complex. Disrupting FOXM1, AXL, and eEF2K signaling inhibits the proliferation, migration, invasion, and spheroid formation of GBM cells while inducing both apoptosis and ferroptosis. Moreover, their TMZ-combined genetic knockdown enhances TMZ efficacy, supporting a multitargeted therapeutic approach to counter GBM’s resistance mechanisms.

However, elucidating the precise interfaces by which FOXM1, AXL, and eEF2K interact (e.g., direct binding domains, phosphorylation events, transcriptional regulation, or even miRNAs-driven regulation) will clarify whether small-molecule inhibitors or genetic inhibitors like miRNAs can disrupt this complex. Moreover, our findings are currently limited to in vitro studies, and given these findings, further validation in in vivo GBM orthotopic xenografts and patient-derived tumor models are warranted. Combinatorial regimens involving TMZ plus an inhibitor cocktail for FOXM1, AXL, and eEF2K—or dual inhibitors targeting key proteins in the complex—might yield improved therapeutic efficacy.

## 5. Materials and Methods

### 5.1. Cell Lines and Cell Culture

The human glioblastoma multiforme cell lines U87, LN229, and U373 were obtained from ATCC. These cell lines were cultured in Dulbecco’s modified eagle medium (DMEM)/F12 medium, supplemented with 10% fetal bovine serum (FBS) and 1% penicillin–streptomycin solution (Sigma, St. Louis, MO, USA). The U87, LN229, and U373 cells were maintained at 37 °C in a humidified incubator with 5% CO_2_.

### 5.2. Patient Dataset Analysis

For GBM tumor and non-tumor patient tissue protein expression comparison analysis, the public Gravendeel dataset from (https://gliovis.bioinfo.cnio.es/, accessed on 7 March 2025) was used [49,50].

### 5.3. siRNA Transfection

Cells were transfected with 100 nM siRNAs (control, eEF2K, AXL, FOXM1; Sigma-Aldrich) using HiPerFect transfection reagent (Qiagen, Germantown, MD, USA). After 72–96 h, cells were harvested for downstream analyses. siRNAs targeting eEF2K (SASI_Hs01_0006-0065), AXL (SASI_Hs01_0004-7869), and FOXM1 (SASI_Hs01_0024-3977-0065) were purchased from Sigma-Aldrich (St. Louis, MO, USA). The mission universal negative control siRNA was used as the control for siRNA treatment. U87 and LN229 cells were seeded at a density of 1 to 1.25 × 10^5^ cells/well in 6-well plates. After 24 h, cells were treated with 100 nM siRNA in DMEM/F12 medium without FBS, facilitated by HiPerFect transfection reagent (Qiagen, Germantown, MD, USA). Following a 4–6-h incubation, 10% FBS was added to each well, and the cells were incubated for 72 h.

### 5.4. Colony Formation Assay

Single-cell suspensions of U87, LN229, and U373 cells were prepared and seeded at a density of 1 × 10^3^ cells/mL into 12-well plates. After 48 h, the cells were treated once with 25 nM siRNA (Control, eEF2K, AXL, FOXM1) and cultured for 10 to 14 days. Colonies were fixed and stained with crystal violet, and colony numbers were quantified using ImageJ (Version 1.54).

### 5.5. Protein Extraction and Western Blot Analysis

siRNA-treated U87 and LN229 cells were collected after 72 h of transfection. The cells were centrifuged at 1500 rpm for 5 min and washed twice with ice-cold phosphate-buffered saline (PBS). After obtaining the pellets, they were incubated with 1% phosphatase inhibitor complex and 1% protease inhibitor complex added to RIPA buffer for 30 min. The lysates were then centrifuged at 13,500 rpm for 15 min at 4 °C. The supernatants were collected and analyzed for protein concentrations using the Pierce™ BCA Protein Assay Kit (Rockford, IL, USA) following the manufacturer’s protocol. Western blotting was performed as follows:

- Membranes were blocked with 5% dry milk in Tris-buffered saline-Tween 20 (TBS-T) and incubated overnight at 4 °C with primary antibodies against eEF2K, GAPDH (Cell Signaling Technology, Danvers, MA, USA), AXL (R&D Systems, Minneapolis, MN, USA), and FOXM1 (Santa Cruz Biotechnology, Dallas, TX, USA).
- After washing with TBS-T, membranes were incubated for 1 h at room temperature with horseradish peroxidase-conjugated anti-rabbit or anti-mouse secondary antibodies (Cell Signaling Technology, Danvers, MA, USA).
- Blots were imaged using the ChemiDoc™ Imaging System (Bio-Rad, Hercules, CA, USA) with chemiluminescent detection using the Immobilon Classico Western HRP Substrate (Millipore Sigma, Burlington, MA, USA).

### 5.6. Co-Immunoprecipitation (IP) Assay

Co-immunoprecipitation was conducted with LN229 cell lines following the manufacturer’s protocol (Abcam, ab206996). LN229 cells were collected with ice-cold lysis buffer and incubated with a rotary mixer for 30 min at 4 °C. The supernatants were collected and incubated overnight with FOXM1 (Santa Cruz Biotechnology), eEF2K (Cell Signaling Technology), and control-IgG mouse (Cell Signaling Technology) antibodies. The complexes were then incubated with A/G Sepharose beads. The complexes were eluted with SDS-PAGE loading buffer and analyzed by Western blotting as input (cell lysate), IgG control IP, and Ab-bound IP.

### 5.7. Spheroid Formation Assay

Single-cell suspensions of U87 and LN229 cells treated with siRNA (control, eEF2K, AXL, FOXM1) were prepared and seeded in ultra-low attachment 6-well plates at a density of 1 × 10^4^ cells/well in 2 mL Complete MammoCult Medium (StemCell Technologies, Vancouver, BC, Canada), with duplicates. The evolution of spheroids was captured from three random representative areas by an inverted microscope (EVOS^®^ FL) at 4× magnification every 24 h. On day 5, spheroids with a diameter > 70 μm in each well were counted to measure the number of spheroids.

### 5.8. Cell Migration Assay

The in vitro wound-healing scratch assay was used to measure cell motility and migration. U87 and LN229 cells were seeded at a density of 1 × 10^5^ cells/well in 6-well plates. After 24 h, siRNA transfections (eEF2K, AXL, FOXM1) were performed as previously described. After 72 h, a scratch was created using a sterile 200 μL pipette tip on the treated monolayer cells. Each well was then gently washed with medium to remove detached cells, and fresh medium was added. Cells in the scratched area were observed, and images were taken at 0, 24, and 36 h using the EVOS^TM^ FL microscope. The open area between the two sides of the scratch was measured using ImageJ. Results were calculated as the percentage of the closed area.

### 5.9. In Vitro Matrigel Invasion Assay

U87 and LN229 cells were transfected with 100 nM siRNA (control, eEF2K, AXL, FOXM1). Seventy-two hours after treatment, the cells were collected, and an equal number of cells (5 × 10^5^ cells/chamber for U87 and 8 × 10^5^ cells/chamber for LN229) were seeded in serum-free medium into Matrigel-coated Matrigel invasion chambers (BD Biosciences, San Jose, CA, USA). The cells were allowed to invade for 48 h towards the 10% FBS-supplemented medium in the lower part of the chamber. After incubation, the inserts were fixed with Hema3 fixative (FisherBrand, Pittsburgh, PA, USA). The number of invaded cells was counted using a light microscope from four different fields.

### 5.10. Apoptosis Detection with Annexin V Assay

Annexin V assay was performed to determine apoptosis. U87 and LN229 cells were seeded in 6-well plates and transfected with 100 nM siRNA (control, eEF2K, AXL, FOXM1). After 96 h, cells were collected with the medium in which they were incubated and analyzed by Annexin V/propidium iodide staining following the manufacturer’s protocol (FITC–Annexin V kit; BD Pharmingen, San Diego, CA, USA). FITC-labeled cells were analyzed by flow cytometry at the Houston Methodist Research Institute, Flow Cytometry Core. For the siRNA and TMZ combination assays, LN229 and U87 cells were transfected with eEF2K, AXL, and FOXM1 siRNA (100 nM), and TMZ (200 µM and 150 µM) was added after 24 h of siRNA treatment. Annexin V assays were performed at 96 h.

### 5.11. Lipid Peroxidation Assay

For the lipid peroxidation assay, BODIPY™ 581/591 C11 (lipid peroxidation sensor) (Invitrogen™, Carlsbad, CA, USA) was used according to the manufacturer’s protocol. U87 and LN229 cells were seeded in 6-well plates and transfected with 100 nM siRNA (control, eEF2K, AXL, FOXM1). After 96 h, cells were collected and incubated with BODIPY. BODIPY-labeled cells were analyzed by flow cytometry at the Houston Methodist Research Institute, Flow Cytometry Core. For the siRNA and TMZ combination assays, LN229 and U87 cells were transfected with eEF2K, AXL, and FOXM1 siRNA (100 nM), and TMZ (200 µM and 150 µM) was added after 24 h of siRNA treatment. Lipid peroxidation assays were performed at 96 h.

### 5.12. Statistical Analysis

Data are shown as mean ± SD. Statistical comparisons were performed using one-way ANOVA with post hoc tests for multiple comparisons. *p* < 0.05 was considered statistically significant. GraphPad Prism (v9.5.1) was used for data analysis.

## Data Availability

The datasets generated and/or analyzed during the current study are available from the corresponding author on reasonable request.

## Supplementary Materials

The following supporting information can be downloaded at: https://www.mdpi.com/article/10.3390/ijms26146792/s1.

## Abbreviations

The following abbreviations are used in this manuscript:

GBM	Glioblastoma Multiforme
eEF2K	Eukaryotic Elongation Factor 2 Kinase
FOXM1	Forkhead-box protein M1
TMZ	Temozolomide
