# Supplementary material for: Inhibition of FOXM1 Leads to Suppression of Cell Proliferation, Migration, and Invasion Through AXL/eEF2 Kinase Signaling and Induces Apoptosis and Ferroptosis in GBM Cells
Source: Int J Mol Sci. 2025 Jul 15;26(14):6792. doi: 10.3390/ijms26146792 (PMC12296191; doi:10.3390/ijms26146792)
Supplement: Supplementary file 1 [file ijms-26-06792-s001.zip › ijms-3709497-supplementary.pdf]

Supplementary Materials

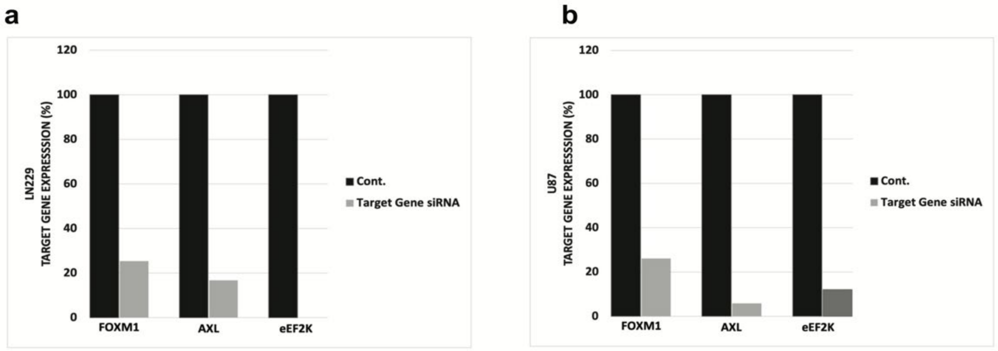

Supplementary Figure S1. *siRNA knockdown efficiency.* (**a**,**b**) FOXM1, AXL and eEF2K siRNAs effectively suppressed its target oncogene expression. Their target gene expressions were observed by less than 30%. The knockdown efficiency was evaluated by Western blot analysis.
